# The clinical significance of HPIP and the associated prognosis in cervical cancer

**DOI:** 10.18632/oncotarget.19607

**Published:** 2017-07-26

**Authors:** Fanling Meng, Haixia Liu, Shuang Liu, Rong Ma

**Affiliations:** ^1^ Department of Gynecology, Harbin Medical University Cancer Hospital, Harbin, China

**Keywords:** hematopoietic pre-B-cell leukemia transcription factor-interacting protein, HPIP, cervical cancer, metastasis, prognosis

## Abstract

Hematopoietic pre-B-cell leukemia transcription factor-interacting protein (HPIP), is known to promote tumor development and metastasis. However its role in cervical cancer remains unknown. The purpose of this study was to investigate the clinical significance of HPIP expression and the prognosis of patients with cervical cancer. Fresh frozen tissues from 10 samples of cervical cancer and 8normal cervical tissues were analyzed for HPIP expression using real-time reverse transcription PCR and Western blot analysis. A total of 129 paraffin-embedded surgical specimens from patients with CC were collected for an immunohistochemistry assay to measure HPIP expression. Correlations of HPIP expression with clinicopathological factors and prognosis of patients with cervical cancer were analyzed. The HPIP expression at both the mRNA and protein levels was significantly higher in cervical cancer tissues than in normal cervical tissues (*P*<0.001). HPIP overexpression was significantly associated with high FIGO stage (*P*=0.005), Histological grade (*P*<0.001), Ascular tumor embolus (*P*=0.004), Iinterstitial infiltration (*P*<0.001), Tumor size (*P*=0.001) and Lymph node metastasis (*P*=0.005). Moreover, results revealed that HPIP expression was an independently prognostic factor for both overall survival [hazard ratio (HR): 8.874; 95% CI: 1.186–66.393; *P*=0.033] and disease-free survival [(HR): 11.523; 95% CI: 1.531–86.746; *P*=0.018] in patients with cervical cancer. The present study provides evidence that HPIP predicts metastasis and poor survival, highlighting its potential function as a therapeutic target for cervical cancer.

## INTRODUCTION

Cervical cancer comprises almost 12% of all female cancers, making it the fourth most common cancer among women worldwide. Specifically, 80% of cervical cancer cases occur in developing countries, and approximately 70% are identified as an advanced disease [1]. A recent study suggests that 61,691 patients were newly diagnosed with CC in China in 2012, accounting for 12% of the new CC cases worldwide [2]. Therefore, the identification of novel cancer biomarkers that can predict the treatment response and prognosis of CC is urgently needed. More importantly, the discovery of new therapeutic targets with high specificity is crucial, which can be accomplished by a better understanding of the molecular mechanisms underlying the carcinogenesis and metastasis of CC.

*HPIP* (Hematopoietic pre-B-cell leukemia transcription factor-interacting protein) is an oncogene that is overexpressed in various human cancers [3]. In addition, HPIP contributes to the development and progression of cancer by promoting proliferation, invasion, metastasis and chemoresistance, which are all hallmarks of aggressive cancer [4]. Our previous studies demonstrated that HPIP overexpression is an independent predictor of chemotherapy resistance and epithelial ovarian carcinoma prognosis [5, 6]. However, the expression of HPIP as a predictive biomarker for clinical significance has not been investigated in patients with cervical cancer.

This study aimed to identify the relationships between HPIP expression and the clinical pathological factors in patients with CC, with the intention of determining whether HPIP could be a biomarker that may predict the metastasis and the prognosis of CC patients.

## RESULTS

### HPIP overexpression in cervical cancer tissues

**Figure 1:**
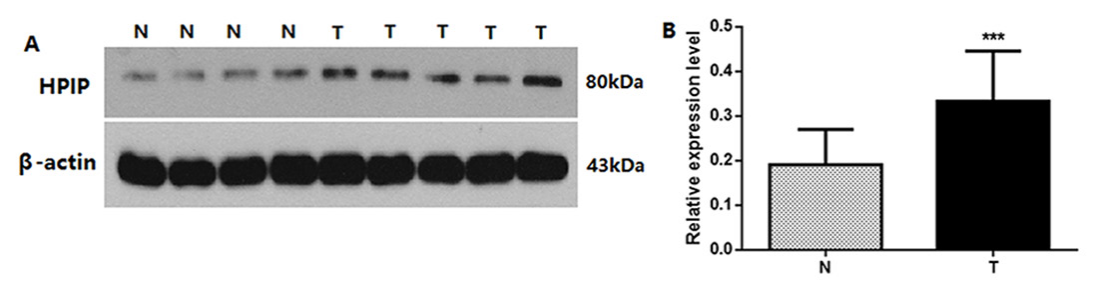
**(A)** Protein samples obtained from frozen normal cervical tissues (N) and cervical cancer tissues (T) were analyzed by Western blot analysis. The levels of β-actin were used as an internal control. **(B)** Histogram of pooled data from N (n=8) and CCs (n=10). HPIP expression was elevated in CCs compared with N. The data are presented as mean±s. d., (*** *P*<0.001).

Western blotting was performed to evaluate differences in HPIP protein expression in cervical cancers and normal cervical tissues. Specifically, HPIP expression was low in normal tissues, whereas high expression was found in tissues from patients with cervical cancer (*P*<0.001, Figure 1).

**Figure 2:**
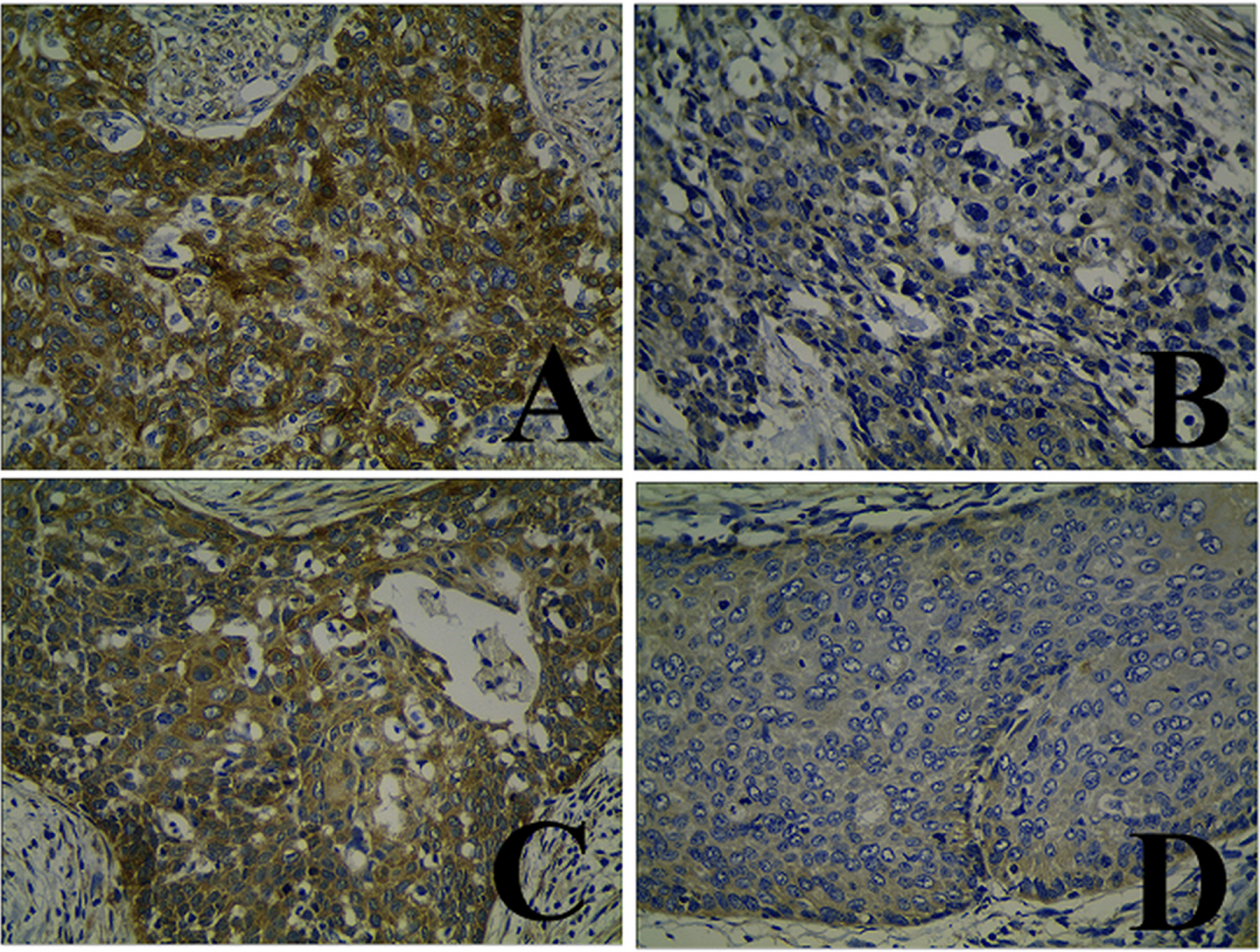
Immunohistochemical staining of HPIP in cervical cancer (CC) specimens **(A** and **C)** High expression of HPIP in CCs. **(B** and **D)** Low expression of HPIP in CCs.

The immunohistochemistry analysis showed that HPIP expression was localized in the cytoplasm of tumor cells (Figure 2). Of the 129 CC cases, 35 (27.1%) and 94 (72.9%) showed low and high HPIP expression, respectively.

**Figure 3:**
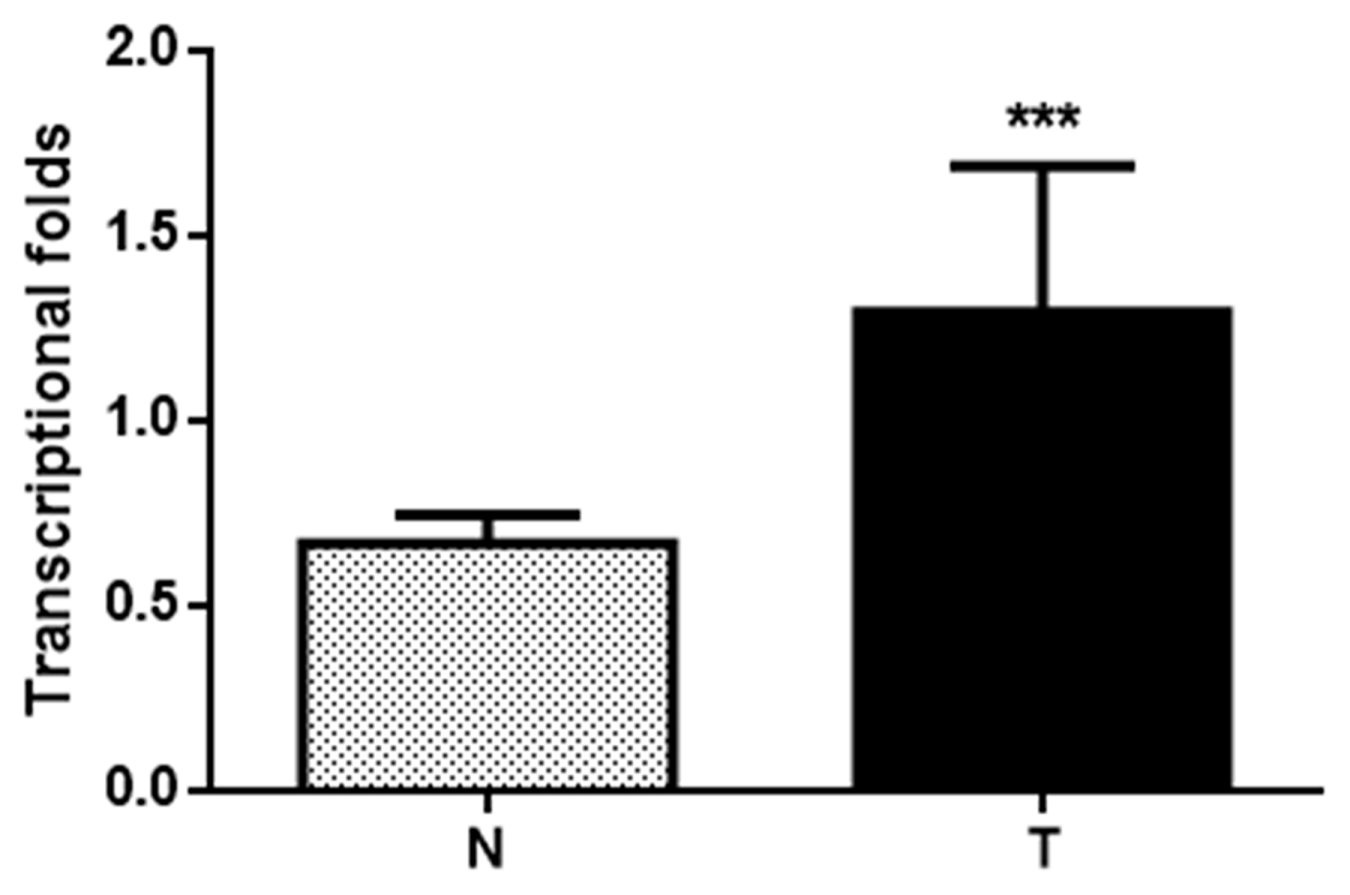
Histogram of HPIP mRNA expression in normal cervical tissues and cervical cancer tissues (N, normal cervical tissues; T, cervical cancer tissues) The levels of β-actin were used as an internal control, and the HPIP mRNA expression was calculated by 2^-∆∆Ct^ method. HPIP mRNA expression was elevated in CCs compared with normal cervical tissues. The data are presented as mean±s. d, (*** *P*<0.001).

The mean expression level of HPIP mRNA was significantly higher in cervical cancer tissues than in normal cervical tissues (*P*<0.001, Figure 3).

### Association between HPIP overexpression and clinical pathological factors in CC

High HPIP expression was significantly associated with FIGO stage (*P*=0.005), ascular tumor embolus (*P*=0.004), interstitial infiltration (*P*<0.001), tumor size (*P*=0.001), histological grade (*P*<0.001), and lymph node metastasis (*P*=0.005). However, HPIP expression did not correlate with age (*P*=0.681), histologic type (*P*=0.113) and preoperative serum SCC level (*P*=0.411) (Table 1).

**Table 1:** Association analyses between the expression levels of HPIP and the clinicopathological characteristics of CC

Variables	Patients n	HPIP	expression	*P*^a^
		Low	High	
All cases				
Age(years)				
≤55	45	11	34	*P*=0.681
>55	84	24	60	
FIGO stage				
I	73	27	46	*P*=0.005
II	56	8	48	
Histological grade				
G1	83	31	52	*P*<0.001
G2/G3	46	4	42	
Histological type				
SCC	116	29	87	*P*=0.113
Adenocarcinoma	13	6	7	
SCC (Uml^-1^)				
≤1.5	84	25	59	*P*=0.411
>1.5	45	10	35	
Ascular tumor embolus				
No	94	32	62	*P*=0.004
Yes	34	3	31	
Iinterstitial infiltration				
≤1/2	68	29	39	*P*<0.001
>1/2	61	6	55	
Tumor size				
≤4cm	74	28	46	*P*=0.001
>4cm	55	7	48	
Lymph node metastasis				
No	106	34	72	*P*=0.005
Yes	23	1	22	

HPIP, hematopoietic pre-B cell leukemia transcription factor (PBX)-interacting protein; FIGO, International Federation of Gynecology and Obstetrics; G1, well differentiated; G2, moderately differentiated; G3, poorly differentiated; SCC, squamous cells carcinoma; EOC, epithelial ovarian cancer; ^a^ chi-square test.

### Prognostic value of HPIP expression

**Figure 4:**
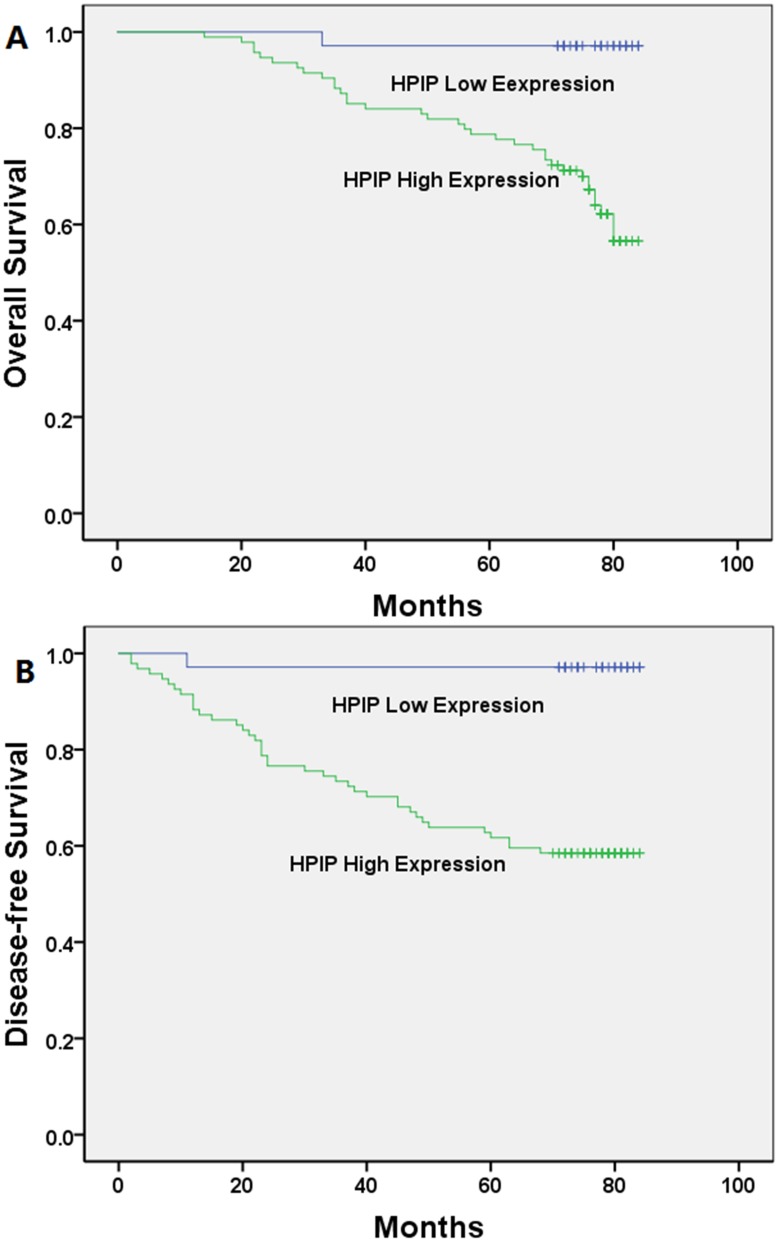
Kaplan-Meier analysis of overall survival and disease-free survival related to the expression of HPIP Patients with high expression of HPIP had a poorer prognosis than those of patients with low expression of HPIP. **(A)** Overall survival curves of the CC according to their HPIP expression status, *P*<0.001; **(B)** disease-free survival curves of the CC patients according to their HPIP expression status, *P*<0.001.

A univariate Kaplan–Meier analysis showed that high HPIP expression was associated with poor OS or DFS in patients with CC (Figure 4; Table 2; P< 0.001).

**Table 2:** Univariate survival analysis of OS and DFS in 129 patients with CC

Variables	n	OS		*P*^a^	DFS		*P*^a^
		Mean±SE (month)	95%CI		Mean±SE (month)	95%CI	
Age(years)							
≤55	45	76±3	71-81	*P*=0.711	67±4	60-75	*P*=0.804
>55	84	74±2	70-78		66±3	60-73	
FIGO stage							
I	73	77±2	73-81	*P*=0.084	71±3	66-77	*P*=0.027
II	56	70±3	65-76		60±4	52-68	
Histological grade							
G1	83	79±2	75-82	*P*=0.001	73±3	68-78	*P*<0.001
G2/ G3	46	67±3	60-73		54±5	45-63	
Histological type							
SCC	116	75±5	66-84	*P*=0.535	37±2	33-40	*P*=0.915
Adenocarcinoma	13	74±2	71-78		35±3	30-41	
SCC (Uml^-1^)							
≤1.5	84	75±2	71-79	*P*=0.876	66±3	60-72	*P*=0.710
>1.5	45	74±3	68-79		68±4	60-76	
Ascular tumor embolus							
No	94	77±2	73-80	*P*=0.001	71±3	66-76	*P*=0.004
Yes	34	67±3	60-73		54±5	44-64	
Iinterstitial infiltration							
≤1/2	68	80±2	77-83	*P*=0.001	74±3	69-79	*P*=0.001
>1/2	61	69±3	63-74		58±4	51-66	
Tumor size							
≤4cm	74	81±1	78-83	*P*<0.001	78±2	74-82	*P*<0.001
>4cm	55	65±3	59-71		51±4	43-59	
lymph node metastasis							
No	106	76±2	72-79	*P*=0.017	70±3	65-75	*P*=0.002
Yes	23	68±4	60-77		52±6	40-64	
HPIP expression							
Low expression	106	83±1	80-85	*P*<0.001	82±2	78-86	*P*<0.001
High expression	23	71±2	67-76		61±3	62-72	

FIGO, International Federation of Gynecology and Obstetrics; G1, well differentiated; G2, moderately differentiated; G3, poorly differentiated; HPIP, hematopoietic pre-B cell leukemia transcription factor (PBX)-interacting protein; OS, overall survival; DFS, disease-free survival; EOC, epithelial ovarian cancer; ^a^ log-rank test.

Furthermore, a multivariate Cox regression analysis demonstrated that HPIP expression was an independent prognostic factor for OS or DFS in patients with CC (Figure 4; Table 3; P< 0.05).

**Table 3:** Multivariate survival analysis of OS and DFS in patients with CC

Variables	OS			DFS		
	Exp(B)	95%CI	*P*^a^	Exp(B)	95%CI	*P*^a^
Age	2.202	0.971-4.993	*P*=0.059	2.097	0.960-4.579	*P*=0.063
FIGO stage	1.135	0.566-2.276	*P*=0.722	1.434	0.743-2.770	*P*=0.283
Histological grade	1.774	0.810-3.884	*P*=0.152	2.173	1.031-4.579	*P*=0.041
Histological type	2.259	0.590-8.653	*P*=0.234	3.676	0.920-14.695	*P*=0.066
SCC (Uml^-1^)	0.732	0.315-1.700	*P*=0.468	0.520	0.229-1.177	*P*=0.117
Ascular tumor embolus	1.648	0.773-3.513	*P*=0.196	1.262	0.633-2.518	*P*=0.509
Iinterstitial infiltration	1.776	0.733-4.304	*P*=0.203	1.828	0.768-4.350	*P*<0.173
HPIP expression	8.874	1.186-66.393	*P*=0.033	11.523	1.531-86.746	*P*=0.018
Tumor size	3.444	1.559-7.612	*P*=0.002	4.633	2.139-10.032	*P*<0.001
lymph node metastasis	0.985	0.400-2.428	*P*=0.974	1.288	0.551-3.015	*P*=0.559

FIGO, International Federation of Gynecology and Obstetrics; HPIP, hematopoietic pre-B cell leukemia transcription factor (PBX)-interacting protein; OS, overall survival; DFS, disease-free survival; CI, confidence interval; CC, cervical cancer; ^a^ Cox regression test.

## DISCUSSION

To the best of our knowledge, the present study is the first to assess the status of HPIP expression at both the mRNA and protein levels as well as the first to assess the association of clinicopathological parameters and prognostic significance of HPIP protein expression in CC. Interestingly, we found that high HPIP expression was associated with unfavorable biological behavior and poor prognosis.

HPIP fulfills many functions and serves as an important molecular biomarker in carcinogenesis. The protein expression of HPIP has been explored and is correlated with tumorigenesis and metastasis in various cancers, such as breast cancer [10], lung cancer [11], head-and-neck squamous cell carcinoma [12], thyroid cancer [13], gastric cancer [14], colorectal cancer [15] and oral carcinoma [15]. Our previous studies also indicated that the overexpression of HPIP is associated with poor prognosis in a large portion of epithelial ovarian carcinoma [6]. These findings suggest that HPIP is an important proto-oncogene during the cancer development. However, the expression of HPIP protein and its association with important prognostic factors in cervical cancer remain unknown.

In the present work, Western blotting revealed that HPIP protein expression was low in normal tissues and high in cervical cancers. HPIP over-expression in CC was evident at both the protein and mRNA levels by real-time reverse transcription PCR and Western blot analysis. Furthermore, immunohistochemistry analysis revealed that high HPIP expression was significantly correlated withFIGO stage, ascular tumor embolus, interstitial infiltration, tumor size, histological grade, and lymph node metastasis. However, HPIP expression did not correlate with age, histologic type and preoperative serum SCC level. Data from the Kaplan–Meier method and log-rank test also demonstrated that the patients with high HPIP expression exhibited significantly poor overall survival and disease free survival. Multivariate analysis demonstrated that HPIP expression was an independent prognostic factor for both overall survival and disease free survival in CC patients. These results suggest that high HPIP expression promotes CC progression and is significantly associated with an independent poor prognostic factor.

To date, several studies have elucidated the molecular mechanisms by which HPIP promotes cancer development. Shi et al. revealed that demonstrated that HPIP silencing suppressed TGF-β1-induced EMT in lung cancer cells by inhibiting Smad2 activation [16]. Bugide S et al. found that HPIP can promote the migration, invasion and EMT of ovarian cancer cells and induces EMT in these cells via activation of the PI3K/AKT pathway [17]. Data from another study demonstrated that knockdown of HPIP inhibited NSCLC cell proliferation and invasion through suppression of the Sonic hedgehog signaling pathway [4]. In addition, the estrogen receptor-interacting protein HPIP may increase estrogen-responsive gene expression through activation of MAPK and AKT signal pathway [18]. These data indicate that HPIP may serve as a potential target for anticancer therapies due to its significant role in cancer and that specific inhibitors of HPIP may be therapeutically used in various cancers.

The limitation of predicting cancer metastasis emphasizes the importance of identifying biomarkers for cancer progression and encourages further investigation. There are also some limitations to the current study. First, only a relatively small number of samples were available for statistical analysis, suggesting that a much larger study is needed to effectively validate our conclusion. Second, our study was limited to several known clinico-pathological factors that were examined for association with HPIP expression. More studies are needed to evaluate HPIP expression with other risk factors for cervical cancer, such as human papillomavirus (HPV) infection.Second, our study was limited to the assessment of several known clinicopathological factors that were examined for an association with HPIP expression. More studies are needed to evaluate the association of HPIP expression with other risk factors for CC, such as human papillomavirus (HPV) infection.

In conclusion, we have shown that HPIP is overexpressed in a large proportion of cervical carcinomas and that the high HPIP expression can be correlated with disease progression and poor prognosis. These results suggest that HPIP may be an attractive therapeutic target for the treatment of cervical cancer. However, these findings remain to be confirmed by a much larger study.

## MATERIALS AND METHODS

### Patient population

Paraffin-embedded tissue samples were collected from 129 patients with CC who were diagnosed between October 2006 and October 2007 in the Harbin Medical University Cancer Hospital.

All patients underwent radical hysterectomy and pelvic lymphadenectomy. In addition, fresh tissues from 18 patients, including tumour tissues (n=10) and normal tissues (n=8), were collected and stored at −80°C immediately after resection to extract the protein and RNA. Patients with normal cervical tissues underwent hysterectomy with oophorectomy for benign uterine disease.

The clinical and pathological characteristics of the patients, including age at diagnosis, histological grade, lymph node metastasis, ascular tumor embolus, SCC, interstitial infiltration, histologic type, tumor size and FIGO stage, are described in Table 1. The tumor stages were evaluated following the International Federation of Gynecology and Obstetrics (FIGO) staging system [7]. Histological typing was classified according to World Health Organization classification standards [8].

All patients with CC were monitored for survival analysis until 31 October 2013 (mean, 70 months; range, 14–84 months). This study was approved by the Ethical Committee of the Affiliated Tumor Hospital of Harbin Medical University.

### Immunohistochemistry and evaluation

Immunohistochemistry was performed using an anti-HPIP antibody (Abcam, USA) following our previously published standard method [5]. Briefly, all sections were deparaffinized in xylene and rehydrated with distilled water through a graded series of ethanol solutions. Antigen retrieval was performed by boiling the sections under pressure for 4 min and then immersed them in 0.01 mol/L citrate buffer (pH 6.0), after cooling down to room temperature.

The sections were incubated overnight with primary rabbit anti-HPIP antibodies (Abcam, Cambridge, MA, USA), with a diluted ratio of 1:200. Biotinylated anti-rabbit antibodies from Beijing Zhongshan Golden Bridge Biotechnology Company (China) were used as the second antibody. HPIP expression was visualised using 3,3’-diaminobenzidine tetrahydrochloride (DAB) and the sections were subsequently counterstained in Mayer’s haematoxylin. At last, all sections were dehydrated through a graded series of alcohols and xylene before being mounted under a cover slip. A paraffin-embedded block of a proven epithelial ovarian carcinoma was used as a positive control. We used a paraffin-embedded block of a proven ovarian cancer as a positive control and the negative control slides were stained with rabbit serum instead of primary antibodies.

The HPIP protein expression levels were semi-quantitatively classified based on the total combined positively stained tumor cell percentage and staining intensity scores, as previously described. The staining intensity score plus the percentage of positive staining was used to evaluate the expression levels, where 0-2 indicated low expression and 3-12 indicated high expression.

### Western blot analysis

Eighteen frozen tissue samples were homogenized in RIPA buffer (Abcam, Cambridge, MA, USA) containing a 1% protease inhibitor cocktail. The protein expression levels were evaluated by Western blotting with an anti-HPIP antibody (1:500, Abcam, Cambridge, MA, USA) according to the manufacturer’s instructions. The Western blotting was performed as previously reported [9]. The protein was extracted from tissues by using RIPA (Beyotime) and protein concentration was determined by Bradford assay using BSA. Equal quantities of protein were separated electrophoretically on 10% sodium dodecyl sulfate polyacrylamide gel and transferred onto polyvinylidene difluoride membranes (Millipore, Billerica, MA, USA). Primary antibodies, anti-HPIP (1:500, Abcam, Cambridge, MA, USA), and anti-β–actin (Santa Cruz Biotechnology, Santa Cruz, CA) were diluted in buffer and incubated at 4°C overnight. The experiment was performed in triplicate.

### Real-time RT-PCR

Total RNA was isolated from cervical cancer tissues and normal cervical tissues (n=18) using TRIzol reagent (Invitrogen Life Technologies, Carlsbad, CA, USA) according to the manufacturer’s protocol. The Superscript III Platinum Kit (Invitrogen) was used to reverse transcribe this total RNA into cDNA. Real-time PCR was performed with SYBR Green Master Mix (TaKaRa, Kyoto, Japan) using the following primers against HPIP: Forward, 5'-CCACCCACTTCTCTCAACTC-3'; Reverse, 5'-GATGAGGCTGCCAGGATA-3'. β-actin served as an internal reference; its expression was analyzed using the following primers: Forward, 5'-CTTAGTTGCGTTACACCCTTTCTTG-3'; Reverse, 5'-CTGTCACCTTCACCGTTCCAGTTT-3'. The experiments were performed in triplicate in the same reaction, and the results of the real-time quantitative RT-PCR experiments were analyzed using the 2^−ΔΔCt^ method.

### Statistical analysis

Student’s t-test was used to compare continuous variables. The association between HPIP expression and clinical pathological factors was assessed using univariate and multivariate logistic regression with covariate adjustment. Survival was calculated using Kaplan–Meier method and evaluated by log-rank test. Cox proportional hazard regression was performed for multivariate analysis of prognostic predictors. All statistical analyses were performed using SPSS 24.0 Software (SPSS, Chicago, IL, USA), and differences were considered significant at *P*<0.05.
